# Age-related somatic mutation burden in human tissues

**DOI:** 10.3389/fragi.2022.1018119

**Published:** 2022-09-21

**Authors:** Peijun Ren, Xiao Dong, Jan Vijg

**Affiliations:** ^1^ Center for Single-Cell Omics, School of Public Health, Shanghai Jiao Tong University School of Medicine, Shanghai, China; ^2^ Department of Genetics, Cell Biology and Development, Institute on the Biology of Aging and Metabolism, University of Minnesota, Minneapolis, MN, United States; ^3^ Department of Genetics, Albert Einstein College of Medicine, New York City, NY, United States

**Keywords:** somatic mutation, mutation burden, clonal amplification, aging, single-cell sequencing

## Abstract

The genome of multicellular organisms carries the hereditary information necessary for the development of all organs and tissues and to maintain function in adulthood. To ensure the genetic stability of the species, genomes are protected against changes in sequence information. However, genomes are not static. *De novo* mutations in germline cells are passed on to offspring and generate the variation needed in evolution. Moreover, postzygotic mutations occur in all somatic cells during development and aging. These somatic mutations remain limited to the individual, generating tissues that are genome mosaics. Insight into such mutations and their consequences has been limited due to their extremely low abundance, with most mutations unique for each cell. Recent advances in sequencing, including whole genome sequencing at the single-cell level, have now led to the first insights into somatic mutation burdens in human tissues. Here, we will first briefly describe the latest methodology for somatic mutation analysis, then review our current knowledge of somatic mutation burden in human tissues and, finally, briefly discuss the possible functional impact of somatic mutations on the aging process and age-related diseases, including cancer and diseases other than cancer.

## Introduction

Mutations, here defined as changes in genome sequence varying from single nucleotide variants (SNVs) and small insertions and deletions (INDELs) to large structural variations (SVs) and chromosomal changes, are consequences of errors in DNA transactions. For example, SNVs are often due to replication errors (Preston et al., 2010) while SVs can be caused by errors in repairing DNA double-strand breaks (Rodgers and McVey, 2016). Somatic mutations are inevitable because their complete prevention would eliminate the genetic diversity that is the substrate of evolutionary adaptation. It is also physiologically costly, which would lead to fitness loss (Sniegowski et al., 2000). Mutations are irreversible in the absence of a readily available reserve template. Indeed, correction systems are limited to proofreading and DNA mismatch repair during and immediately after replication (Robinson et al., 2021). Hence postzygotic somatic mutations effectively turn tissues into genome mosaics.

The quantitative analysis of somatic mutations is a challenge because they occur more or less randomly and differ from cell to cell. This is why in the past selectable reporter genes in mouse models were used to compare somatic mutations in organs and tissues during aging (Boerrigter et al., 1995). Studies with these mouse models showed that somatic mutation burdens are tissue-specific, increase with age and are far higher than expected based on existing information on germline mutations (Dolle et al., 1997; Dolle et al., 2000). Recently, major advances in sequencing, including single-cell sequencing, opened the possibility to quantitatively study somatic mutations directly in human tissues. Here we will review the latest progress in this field.

## Sequencing approaches to studying somatic mutations

The first methods for the quantitative analysis of somatic mutations were based on whole genome amplification and sequencing of single cells (Gundry et al., 2012; Zong et al., 2012). However, amplification is prone to error and to resolve this problem, methods were developed, including computational methods, to prevent and filter out such amplification errors (Dong et al., 2017; Bohrson et al., 2019).

Somatic mutations were also analyzed in clones derived from single cells, including organoids derived from tissue biopsies (Blokzijl et al., 2016; Franco et al., 2018). Indeed, most tumors are derived from single cells, which makes them suitable surrogates for mutation burdens present in the normal cell that gave rise to the tumor. This is reflected by the observed increase with age of the number of mutations found in tumors (Alexandrov et al., 2015; Milholland et al., 2015).

Somatic mutations can clonally amplify in a mitotically active tissue as a consequence of a fitness advantage or through genetic drift. Such clonally amplified mutations can be detected through bulk sequencing at high depth, and they increase with age (Martincorena et al., 2018). The existence of such mutant clones was first demonstrated in blood and termed clonal hematopoiesis (Busque et al., 2012; Jaiswal et al., 2014). Clonally amplified somatic mutations can also be detected at the RNA level, which has been done for many human tissues using raw RNA-sequencing reads from dbGAP GTEx (Garcia-Nieto et al., 2019; Yizhak et al., 2019).

Finally, somatic mutations can be detected in bulk DNA samples at the single molecule level. The problem of artifacts drowning out true mutations was first addressed by Loeb and co-workers who developed duplex sequencing (Schmitt et al., 2012). In this method, opposite strands of DNA fragments are tagged using unique molecular identifiers (UMIs), sequenced and reconstructed by computational means. True mutations are found at the same position in both strands, while amplification and sequencing errors are found only in one. This method has been greatly improved recently and now allows to interrogate 1 billion random bases per tissue sample across the genome, providing a highly representative picture of the mutational burden, spectra, and signatures as well as the distribution of mutations among genome functional elements (Maslov et al., 2022).

In the next section we will summarize the results obtained with these approaches and the current status of the quantitative analysis of somatic mutations in human tissues as a function of age.

## Tissue-specific somatic mutation burden as a function of age

**FIGURE 1:**
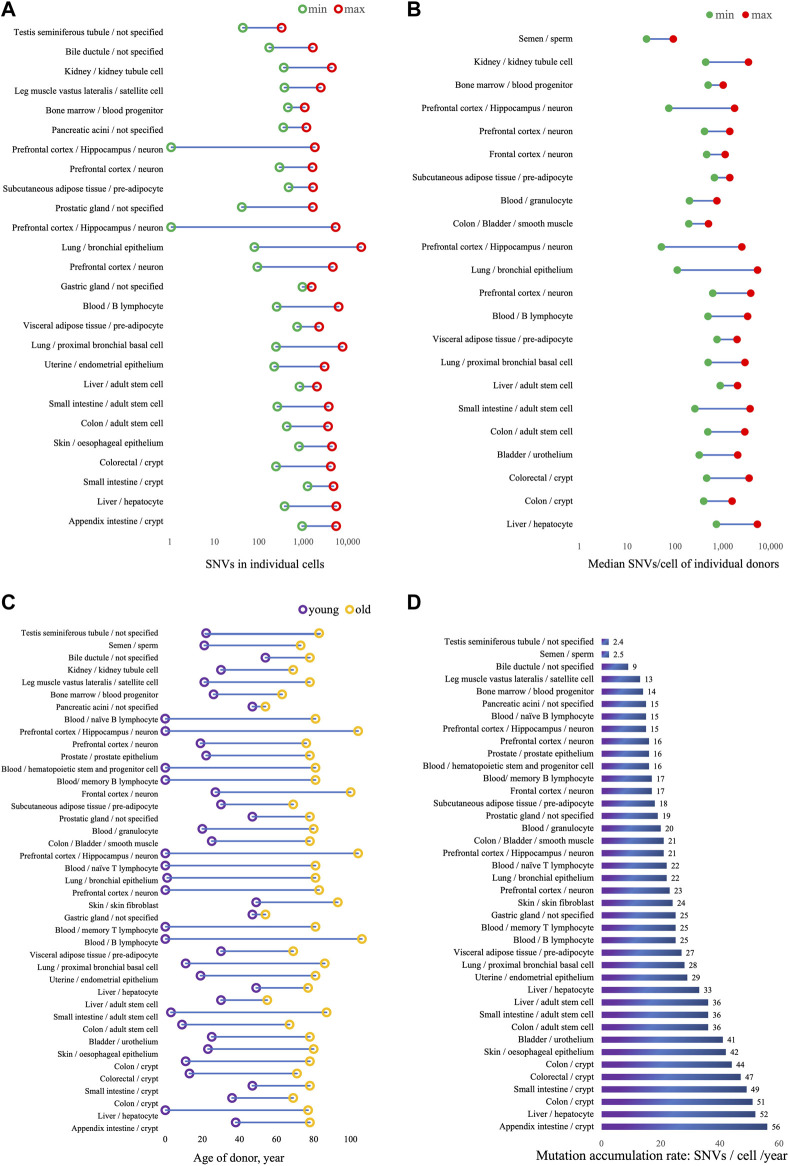
Somatic mutation burden and accumulation rates in normal human tissues. **(A)** Range of SNVs counts detected in all individual cells. SNVs counts are from original data in publication, noted in Table 1, and donor age in not considered. **(B)** Median SNVs per cell in individual donors, calculated from published SNVs counts per cell, donor age in not considered. **(C)** Age range of donors. **(D)** Annual mutation accumulation rate, SNVS/cell/year in individuals.

Somatic mutation burden can vary between cell types, tissues and organs, between individuals, in relation to disease and due to environmental conditions. As increasingly more somatic mutation data are now being collected (Sun et al., 2022), researchers are now able to compare somatic mutation profiles in different tissues and cell types as a function of age. Table 1 lists the results obtained thus far, the results are also visualized in Figure 1. One universal feature of somatic mutations in all normal human tissues is their accumulation with age.

**TABLE 1:** Somatic mutation burden in relation to age in normal human tissues.

Tissue	Cell type	Sequencing method	SNVs/cell^a^	Range of SNVs/cell of individual donors (median)^b^	Donor age	SNVs/cell/year^c^	References
Appendix intestine	Crypt	LCM-seq	883∼5,160	na.	38∼78	∼56	Moore et al. (2021)
Liver	hepatocyte	SCMDA	357∼5,206	691∼4,978	0∼77	∼52^d^	Brazhnik et al. (2020)
Colon	Crypt	NanoSeq	na.	376∼1,473	36∼69	∼51	Abascal et al. (2021)
Small intestine	Crypt	LCM-seq	1,178∼4,502	na.	47∼78	∼49	Moore et al. (2021)
Colorectal	Crypt	LCM-seq	230∼3,907	434∼3,340	13∼71	∼47	Cagan et al. (2022)
Colon	Crypt	LCM-seq	na.	na.	11∼78	∼44	Lee-Six et al. (2019)
Skin	oesophageal epithelium	bulk DNA	749∼4,170	na.	23∼80	∼42	Yokoyama et al. (2019)
Bladder	urothelium	NanoSeq	na.	303∼1,940	25∼78	∼41	Abascal et al. (2021)
Colon	adult stem cell	single-cell colony	400∼3,383	458∼2,733	9∼67	∼36	Blokzijl et al. (2016)
Small intestine	adult stem cell	single-cell colony	245∼3,516	246∼3,516	3∼87	∼36	Blokzijl et al. (2016)
Liver	adult stem cell	single-cell colony	771∼1,919	829∼1,919	30∼55	∼36	Blokzijl et al. (2016)
Liver	hepatocyte	LCM-seq	na.	na.	49∼77	∼33	Brunner et al. (2019)
Uterine	endometrial epithelium	LCM-seq	209∼2,830	na.	19∼81	∼29	Moore et al. (2020)
Lung (non-smoker)	proximal bronchial basal cell	SCMDA	230∼7,203	464∼2,739	11∼86	∼28	Huang et al. (2022)
Visceral adipose tissue	pre-adipocyte	single-cell colony	684∼2,133	718∼1,867	30∼69	∼27	Franco et al. (2019)
Blood mononuclear cells	B lymphocyte	SCMDA	237∼5,862	463∼3,127	0∼106	∼25^e^	Zhang et al. (2019)
Blood mononuclear cells	memory T lymphocyte	single-cell colony	na.	na.	0∼81	∼25	Machado et al. (2022)
Gastric gland	not specified	LCM-seq	904∼1,454	na.	47∼54	∼25	Moore et al. (2021)
Skin	skin fibroblast	single-cell colony	na.	na.	49∼93	∼24	Park et al. (2021)
Prefrontal cortex	neuron^f^	MDA	87∼4,330	584∼3,630	0∼83	∼23	Lodato et al. (2018)
Lung	bronchial epithelium	single-cell colony	75∼18,911	105∼5,033	1∼81	∼22	Yoshida et al. (2020)
Blood mononuclear cells	naïve T lymphocyte	single-cell colony	na.	na.	0∼81	∼22	Machado et al. (2022)
Prefrontal cortex/Hippocampus	neuron^f^	MDA	1∼5,026	49∼2,352	0∼104	∼21	Miller et al. (2022)
Colon/Bladder	smooth muscle	NanoSeq	na.	184∼472	25∼78	∼21	Abascal et al. (2021)
Blood	granulocyte	NanoSeq	na.	189∼711	20∼80	∼20	Abascal et al. (2021)
Prostatic gland	not specified	LCM-seq	39∼1,549	na.	47∼78	∼19	Moore et al. (2021)
Subcutaneous adipose tissue	pre-adipocyte	single-cell colony	438∼1,558	628∼1,316	30∼69	∼18	Franco et al. (2019)
Frontal cortex	Neuron	NanoSeq	na.	432∼1,053	27∼100	∼17	Abascal et al. (2021)
Blood mononuclear cells	memory B lymphocyte	single-cell colony	na.	na.	0∼81	∼17	Machado et al. (2022)
Blood mononuclear cells	hematopoietic stem and progenitor cell	single-cell colony	na.	na.	0∼81	∼16	Machado et al. (2022)
Prostate	prostate epithelium	LCM-seq	na.	na.	22∼78	∼16	Grossmann et al. (2021)
Prefrontal cortex	Neuron	META-CS	273∼1,515	390∼1,318	19∼76	∼16	Xing et al. (2021)
Prefrontal cortex/Hippocampus	neuron^f^	PTA	0∼1,707	70∼1,666	0∼104	∼15	Miller et al. (2022)
Blood mononuclear cells	naïve B lymphocyte	single-cell colony	na.	na.	0∼81	∼15	Machado et al. (2022)
Pancreatic acini	not specified	LCM-seq	333∼1,101	na.	47∼54	∼15	Moore et al. (2021)
Bone marrow	blood progenitor	single-cell colony	423∼1,018	464∼964	26∼63	∼14	Osorio et al. (2018)
Leg muscle vastus lateralis	satellite cell	single-cell colony	354∼2,323	na.	21∼78	∼13	Franco et al. (2018)
Kidney	kidney tubule cell	single-cell colony	342∼4,132	412∼3,252	30∼69	12/57^g^	Franco et al. (2019)
Bile ductule	not specified	LCM-seq	161∼1,540	na.	54∼78	∼9	Moore et al. (2021)
Semen	Sperm	NanoSeq	na.	24∼87	21∼73	∼2.5	Abascal et al. (2021)
Testis seminiferous tubule	not specified	LCM-seq	41∼306	na.	22∼83	∼2.4	Moore et al. (2021)

a Range of SNVs counts detected in all individual cells, donor age in not considered. Outlier numbers are excluded.

b Median of SNVs per cell in individual donors, calculated from published SNVs counts per cell, donor age in not considered.

c Annual SNV accumulation rate. Unless otherwise specified, numbers are original data from references.

d, e Linear fitted from published SNVs counts per cell with the age of donors.

f Neuron from neurotypical donors.

g Two kidney donors exhibited high variation.

LCM-seq: Laser-capture microdissection and low-input DNA sequencing (Ellis et al., 2021); SCMDA: single-cell multiple displacement amplification (Dong et al., 2017); single-cell colony: clonal expansion of single cells by in vitro culture; MDA: multiple displacement amplification (Spits et al., 2006); META-CS: multiplexed end-tagging amplification of complementary strands; PTA: primary template-directed amplification.

Using single-cell approaches, somatic mutation burdens have been found to be substantial and to increase with age, in human brain (Lodato et al., 2018), B lymphocytes (Zhang et al., 2019), liver (Brazhnik et al., 2020), and lung (Huang et al., 2022). The highest numbers of mutations were found in liver, possibly because of the role of this organ in detoxification. In normal hepatocytes, median SNV number per cell was found to be 1,222 ± 855 in subjects less than 36 years and to increase to 4,054 ± 1,168 SNVs per cell in those over 46 years (Brazhnik et al., 2020). In human proximal bronchial basal cells from non-smokers, the SNV number was 464 per cell in an 11-year-old and increased to 2,739 per cell at age 86, a mutation accumulation rate of ∼29 SNVs per cell per year (Huang et al., 2022). In healthy human B lymphocytes, the somatic mutation frequency in newborn was less than 500 per cell and increased to well over 3,000 per cell in centenarians (Zhang et al., 2019).

Single-cell approaches have been applied to assessing somatic mutation burden in neurons from both normal subjects and those suffering from neurodegenerative disease, including Alzheimer’s disease (Lodato et al., 2015; Lodato et al., 2018; Miller et al., 2022). In normal neurons from prefrontal cortex and hippocampus, SNV counts in a 0.4-year-old donor was less than 100, increasing to more than 2,000 in people over 80, mutations accumulating at a rate of 16∼21 SNVs/cell/year.

Somatic mutation frequencies have also been assessed in clones derived from single cells. This is very similar to the use of tumors for that purpose, which also derive from a single cell. Indeed, the grown clone or tumor should contain all mutations present in the original single cell. Whole genome sequencing of DNA from these clones circumvents the need for whole genome amplification, which is error-prone. However, clonal amplification of cells in culture is time-consuming and can only be done with cells that can be expanded, especially mitotically active cells, for example, stem or progenitor cells. Taking this approach, Franco et al. showed that in human skeletal muscle satellite cells mutations accumulate with age at a rate of 13 per genome per year (Franco et al., 2018). Accumulation of somatic mutations were also detected in pre-adipocytes isolated from different parts of the kidney with different accumulation rates, 18 and 27 SNVs/cell/year in pre-adipocyte from subcutaneous and visceral adipose tissue, respectively (Franco et al., 2019). Figure 1; 

For human lung, whole genome sequencing of clones derived from single bronchial epithelial cells of non-smoking donors, also showed that single base substitutions increased significantly with age, at an estimated rate of 22 mutations per cell per year (Yoshida et al., 2020). Osorio et al. reported lifelong mutation accumulation in human hematopoietic stem and progenitor cells at the rate of 14 mutations per year per cell. They detected around 450 SNVs per cells in a 26-year-old donor and around 1,000 SNVs per cell in people in their sixties (Osorio et al., 2018). Machado et al. (2022) found SNVs to increase in memory T lymphocytes at a rate of ∼25 per cell per year, a little faster than in naïve T lymphocytes where the rate was 22 per cell per year. Somatic mutation rates in memory (17 SNVs/cell/year) and naïve B lymphocytes (15 SNVs/cell/year) were found to be close to the rate in hematopoietic stem and progenitor cell (16 SNVs/cell/year).

Somatic mutation accumulation with age has also been derived from an observed increase in naturally clonally amplified mutations within human blood or tissues. Also in this case these clones can act as surrogates for mutation accumulation in the cell from which the clone originated. LCM-seq (laser-capture microdissection and low-input DNA sequencing) has been used to capture multiple small clones of no more than hundreds of cells in solid tissue and generate sequencing libraries from nanograms of input DNA (Martincorena et al., 2018; Ellis et al., 2021). The number of such clones increase with age and it is possible to estimate the mean number of mutations per cell in each individual (Martincorena et al., 2018).

Moore et al. compared mutational landscape in multiple samples from the same individuals, and quantified tissue-specific somatic mutation burden and accumulation rate. This study included 14 donors aged from 22 to 83 years, with 22 macroscopically normal tissues and organs collected from the same donor (Moore et al., 2021). The lowest mutation accumulation rate was identified in spermatogonia, i.e., 2.38 SNVs per year, confirming the lower germline mutation rate as compared to somatic mutations (Milholland et al., 2017). Colonic crypt exhibited the highest mutation accumulation rates of 49∼56 SNVs per year, 27-fold higher than in seminiferous tubules. Mutation accumulating rates detected in this study in other tissues were ∼25 in gastric gland, ∼19 in prostatic gland, ∼15 in pancreatic acini and ∼9 in bile ductule. The high mutational burden and accumulation rate in colonic crypt was also observed in other studies using similar methods (Lee-Six et al., 2019; Cagan et al., 2022).

Clonally amplified somatic mutations can also be detected from RNA-seq data. For that purpose, Yizhak et al. (2019) used the Genotype–Tissue Expression (GTEx) data set generated from over 30 normal primary tissues from hundreds of healthy individuals. They found multiple somatic variants, confirming that macroscopic “mutant” clones occur in many if not all normal tissues. They also found an age-related increase in somatic mutations and confirmed that sun-exposed skin, esophagus, and lung have a higher mutation burden than other tested tissues.

All the above approaches require extensive sequencing, often at high depth. For single-cell or single-clone sequencing, multiple cells/clones need to be sequenced for each individual to obtain representative mutation frequencies. By contrast, single molecule sequencing directly from bulk DNA achieves detection of somatic mutations at relatively low sequencing cost. To avoid sequencing errors, the main problem in detecting mutations directly in a DNA sample, single-molecule sequencing uses random barcodes or unique molecular identifiers (UMIs) to create single-molecule-derived reads. As described earlier, amplification artifacts are ruled out by accepting as true only those mutations occurring on each complementary strand opposite each other at the same position. Called Duplex-seq, the original method only allowed to evaluate very small targets, such as mitochondrial DNA (Schmitt et al., 2012). However, more recently other, more efficient variants of the same principle have been developed. For example, Abascal et al. (2021) developed Nanoseq and applied it to study somatic mutations in non-dividing cells across several tissues. The results were very similar to the results obtained by Moore *et al* using LCM mini bulk as mentioned above (Moore et al., 2021) The lowest mutation accumulation rate of 2.5 SNVs/cell/year was identified in sperm, with the highest in colonic crypts of 51 SNVs/cell/year. SNV accumulation rate in neurons was 17 per cell per year, close to the rate detected by other methods as described above. Mutation accumulation rates in urothelium of bladder, smooth muscle cell in colon and bladder, and granulocyte in blood identified in this study were ∼41, ∼21 and ∼20 SNVs/cell/year, respectively.

In summary, there is now absolute consensus that somatic mutations accumulate with age in many if not all human tissues, independent of the method used for mutation evaluation. This fully confirmed results obtained for the mouse using the aforementioned transgenic reporter systems. The mutation frequencies in human tissues and the increase with age were dependent on multiple factors, including environmental mutagens, such as exposure to sun and tobacco smoke. Importantly, as also found in the reporter mice, the accumulation rate of somatic mutations in humans differed significantly among different tissues. In this respect, the two extremes were germ tissue and colorectal crypts (Abascal et al., 2021; Moore et al., 2021). The possible reasons are multiple, but the main one seems to be driven by the length of time needed for a cell or tissue type to function. This is likely why germ tissue has a very low somatic mutation burden and the expendable colonic crypts are tolerant for mutation accumulation. The intestinal epithelium is one of the most rapidly dividing regions of cells in the human body and mutations easily accumulate as replication errors. Also tissues exposed directly to high levels of exogenous genotoxicity harbor heavier mutation burdens, such as liver, skin and lung. Somatic mutations also accumulate with age in hematopoietic cells, albeit at a moderate rate of only 14∼25 SNVs/cell/year (Zhang et al., 2019; Mitchell et al., 2022; Williams et al., 2022). This fairly low rate in spite of continuous mitotic capacity could be related to a low tolerance for mutagenicity due to the extensive cell signaling processes needed in these cells.

## Functional impact of increased mutation burden

Accumulation of somatic mutations will result in intra-tissue genetic heterogeneity, known as genome mosaicism. Thus far, the impact of genome mosaicism on the aging phenotype, other than cancer, remains unclear. Cancer risk increases exponentially as a function of age in both humans and animals through a mechanism of repeated cycles of somatic mutation (often in interaction with germline variants) and selection for a range of characteristics, including growth, tissue invasion, immune suppression and metastasis. Accumulating somatic mutations are likely to play a role in the age-related increase in tumor incidence.

Elsewhere we proposed three possible general mechanisms for a functional impact of age-accumulated somatic mutations: (1) clonal expansion, (2) somatic evolution, and (3) mutational networking (Vijg and Dong, 2020). The first two are based on clonal expansion of a mutation, either because of a selective advantage or genetic drift. They include hyperplastic or neoplastic disease, although mutations that occur early enough can have late-life effects on postmitotic tissues as well (Poduri et al., 2013). The third possibility involves the actual adverse effects of high mutation burden on cell functioning, possibly through destabilization of gene regulatory networks. Genomes are robust and redundancy buffers them against mutations. However, when the mutation burden rises to very high levels, the functional organization of genomes in multiple regulatory sequences serving networks of extensively interacting genes will amplify the effects of mutations.

As mentioned, accumulating evidence is emerging to support the causative role of somatic mutation in diseases other than cancer, especially in degenerative diseases (Poduri et al., 2013; Mustjoki and Young, 2021). Mutation burden in neurons increases with age, which is consistent with what has been found in other normal cell types. Neurodegenerative diseases are associated with elevated mutation burden in single human neurons. Somatic SNVs occur at loci that are expressed in the brain and associated with nervous system function and disease (Lodato et al., 2015). Neurons from patients with Cockayne syndrome, a DNA repair-defective disorder characterized by impaired neuronal development resulting in premature aging, showed a ∼2.3-fold excess of SNVs relative to the expected age-adjusted normal prefrontal cortex rate, while neurons from another DNA repair defective disease, also showing neurological symptoms, Xeroderma Pigmentosum, showed a ∼2.5-fold increase in mutation burden (Lodato et al., 2018). Recent evidence from whole genome sequencing at high depth showed that while mutation load in human brain increased with age, outlier subjects with many more mutations were found associated with old age; this hypermutability was suggested to be due to lineage expansion (Bae et al., 2022). In chronic liver disease, including alcohol-related liver disease and non-alcohol fatty liver disease, burdens of somatic mutations were higher and clonal expansions larger than in normal control subjects, with deleterious mutations found in FOXO1 (Ng et al., 2021). Age-related accumulation of somatic mutations in exons and gene promoters has also been shown to contribute to age-related decline in skeletal muscle function (Franco et al., 2018).

## Conclusion and future prospects

A range of novel, sequencing-based assays have now shown that somatic mutations accumulate in cells across all tissues during the entire human life span. Elevated mutation burden has the potential to impair cellular function, even when most mutations will not affect physiological function. This progress is now greatly improving our understanding of genome mosaicism and its impact on aging and related diseases. A further, drastic reduction in sequencing cost that can be expected in the future will significantly expand our current data sets and will allow to study somatic mutation under many more scenarios with higher accuracy. This would be especially relevant for single-cell sequencing, which remains the gold standard in somatic mutation analysis because it allows determining interactions among mutations in the same genome.

As of yet, isolating single cells from clinical samples is laborious, demands specialized equipment and is expensive. Many assays require fresh tissue samples which cannot always be obtained. Further technical innovation in cell or genome isolation, multi-omics analysis and new, computational pipelines for analyzing variants in relation to their possible epigenetic or transcriptomics endpoints with improved time- and cost-efficiency will broaden the research objects. Besides arising from DNA replication in cell division, erroneous DNA damage repair is another major source for somatic mutation, especially for post-mitotic cells where cell division is absent. Current approaches to detect DNA damage and repair are limited and not accurate. Methods, which could pinpoint the sites of DNA damage and repair in primary human samples, will increase our understanding of how mutations arise.

One critical challenge in our rapidly expanded armamentarium of mutation analysis tools is the lack of robust, quantitative assays for genome structural variation (SV). Such events are much more impactful than SNVs or INDELS, which is why their quantitative assessment in multiple organs and tissues is essential for predicting any functional impact of somatic mutations. Unfortunately, SVs cannot yet be detected in single cells or nuclei. Once we have a full complement of reliable data sets of somatic mutations with age at the single-cell level in all human tissues it will be possible to model the data and test their functional impact on specific functions known to decline with age.
